# Hereditary cancer risk assessment: insights and perspectives for the Next-Generation Sequencing era

**DOI:** 10.1590/1678-4685-GMB-2014-0346

**Published:** 2016-05-13

**Authors:** Israel Gomy, Maria Del Pilar Estevez Diz

**Affiliations:** 1 Instituto de Hematologia e Oncologia de Curitiba, Curitiba, PR, Brazil; 2 Instituto do Câncer do Estado de São Paulo, Faculdade de Medicina da Universidade de São Paulo São Paulo, SP, Brazil

**Keywords:** next-generation sequencing, hereditary cancer, genetic counseling, risk assessment

## Abstract

Hereditary cancer risk assessment is a multidisciplinary and dynamic process, with the purpose of estimating probabilities of germline mutations in cancer susceptibility genes and assessing empiric risks of cancer based on personal and family histories, in order to offer clinical and molecular diagnoses and clinical management based on these risks. Genetic tests are available and most of them are reimbursed by insurance companies, although they are generally not covered by the public health systems of developing countries. More recently, molecular diagnosis of hereditary cancer is feasible through next-generation sequencing (NGS) panels. Here we review the benefits and limitations of NGS technologies in the clinical practice.

## Introduction

Hereditary cancer risk assessment (HCRA) is a quantitative and qualitative process to assess an individual's risk of carrying a certain genetic mutation that predisposes them or their offspring to cancer development. HCRA may be done using mathematical or statistical models incorporating such factors as personal health history, family medical history and ethnic background (Riley *et al*., 2012). The advent of genome sequencing techniques has led to improvements in the multidisciplinary and dynamic processes of estimating probabilities of germline mutations in cancer susceptibility genes and assessing empiric risks of cancer based on personal and family histories. As a result, the molecular diagnosis of hereditary cancer is now possible.

As an integral part of the cancer risk assessment process (Peters and Stopfer, 1996), genetic counseling involves the analysis of pedigrees and risk assessment models to determine whether a family history is suggestive of sporadic, familial or hereditary cancer (Schneider and Garber, 2001). The purpose of cancer genetic counseling is to educate clients about their chances of developing cancer, help them derive meaning from personalized genetic information, and empower them to make educated and informed decisions regarding genetic testing, cancer screening and cancer prevention. Genetic counseling before and after testing is fundamental to the effective implementation of evidence-based protocols, in terms of reducing mortality rates (Trepanier *et al*., 2004).

There is substantial heterogeneity in insurance coverage for genetic testing in developing countries. In Brazil, a middle-income Latin American country, genetic services are sometimes available at private medical offices and clinics. Most genetic tests are reimbursed by private insurance companies, but are not covered by the public health system, Serviço Único de Saúde (Horovitz *et al*., 2013). Care at private healthcare institutions differs from that at public hospitals in terms of the speed of testing and access to complementary exams.

Cancer is essentially a genetic disease, the pathogenesis of which is influenced by both hereditary and environmental factors. Genetic susceptibility and predisposition depend on the penetrance of germline mutations and/or inherited alleles, which may be classified into three groups: high, moderate/intermediate and low penetrance.

High-penetrance alleles predispose an individual to the highest lifetime risks of cancer, frequently more than 10 times the risk in the general population. High-risk alleles often occur in Mendelian autosomal dominant cancer syndromes like in those families with germline mutations in the *BRCA1* and *BRCA2* genes. Mutations in known predisposition genes with high penetrance, such as *BRCA1* and *BRCA2*, account for about 20% of the twofold excess in breast cancer risk among patients' relatives (Anglican Breast Cancer Study Group, 2000). Likewise, about 5% of colorectal cancers can be explained by germline mutations in high-penetrance alleles, such as APC, MLH1 or MSH2.

Alleles with moderate or intermediate penetrance increase the average risk of disease by about two to five times. Although relatively rare in most populations, moderate-penetrance alleles may be common in isolated populations or in populations with high consanguinity rates due to founder effects. Commonly, affected relatives can be identified, but the incomplete penetrance may "skip generations" and can jeopardize the pedigree.

Low-penetrance alleles were discovered mainly through genome-wide association studies (GWAS). They may predispose individuals to cancers at rates slightly higher than those of the general population. This phenomenon may be explained by a polygenic model, in which numerous alleles, each conferring a small genotypic risk (1.5-2.0 times), combine additively or multiplicatively to confer a range of susceptibilities in the population. In this model, individuals carrying few such alleles would be at reduced risk, whereas those with many alleles might suffer a lifetime risk as high as 50% (Pharoah *et al*., 2002). It is estimated that more than 100 common variants with low risk may contribute to cancer susceptibility. Thus, it is very important to identify low-penetrance alleles responsible for genetic susceptibility (Houlston and Peto, 2004). Most of these alleles have an intergenic localization and many neighbor tumor suppressor genes and protoncogenes, possibly affecting their expression.

Despite great efforts to identify new causative variants, most familial cancer risk remains unexplained, the so-called "missing heritability". In breast cancer, for example, *BRCA1* and *BRCA2* are susceptibility genes that account for around 25% of families with hereditary breast cancer (Ford *et al*., 1998). A polygenic mechanism has been proposed, whereby different genetic backgrounds composed of a combination of low-penetrance alleles explain the remaining familial breast cancer risk (Pharoah *et al*., 2002; Seal *et al*., 2006). The first low-penetrance germline mutation associated with familial breast cancer, *CHEK2* 1100delC, was identified in a northern European population, where it conferred a twofold increase in risk (Meijers-Heijboer *et al*., 2002; Vahteristo *et al*., 2002).

Screening of the general population may be necessary to determine those individuals who are most susceptible to cancer. Further testing may be able to stratify these individuals according to genetic risk for disease.

Table 1 summarizes the HCRA process. Three main risk categories can be derived on the basis of patient and family genetic information. In the low-risk (or near-population risk) category, management is based on population screening, genetic tests are generally not cost-effective, and genetic counseling is individual-based; in the moderate-risk group, genetic counseling, genetic testing and management are individual-based; and in the high-risk group, genetic counseling, testing and management are evidence-based and improve survival (Schwartz *et al*., 2009).

**Table 1:** Overview of hereditary cancer risk assessment.

Average risk	High	Moderate/intermediate	Low/near-population
Personal/ family history	Mendelian syndromes	Familial aggregation	Sporadic
Genetic testing	Single gene sequencing*/ NGS panels*/ WGS/ WES	NGS panels/ WGS/ WES	DTC&/ WGS/ SNP genotyping
Genetic counseling	Mandatory	Advisable	Available
Management	Evidence-based*	Individual-based^1^	Not validated

DTC: direct-to-consumer tests; WGS: whole-genome sequencing; WES: whole-exome sequencing.

1 Some evidence-based screening recommendations exist for breast and colorectal cancers.

& Restricted by the US Food and Drug Administration.

## Next-Generation Sequencing

With the publication of the human reference genome and the advent of high-throughput technologies, we have entered the era of next-generation sequencing (NGS). New sequencing technologies have driven the cost of genome sequencing to be close to the much-pursued $ 1,000 genome. Over the last decade, the per-genome cost for sequencing has dropped precipitously, from approximately U$ 70 million in 2002 to nearly U$ 7,000 in 2012. Likewise, the time to generate genomic sequences has decreased, with over 10 billion bases per instrument per day now being possible (Wetterstrand, 2015). These advances in sequencing technology have allowed the elucidation of the underlying genetic bases of several diseases, including Mendelian disorders associated with cancer.

The first use of genome-wide sequencing to identify the genetic cause of a hereditary cancer syndrome used Sanger sequencing and a filter-based approach to compare the coding sequence from germline and tumor cells of a patient with familial pancreatic cancer. In 2013, the whole-genome and whole-exome sequencing of 38 familial pancreatic cancer cases were reported (Roberts and Klein, 2013), describing the aggregation of deleterious germline variants in the *ATM* gene of six pancreatic cancer relatives from two different kindreds. A more recent study (Jaeger *et al*., 2012) used whole-genome sequencing in individuals with hereditary mixed polyposis syndrome to identify a duplication 5′ to *GREM1*, a negative regulator of bone morphogenetic protein (BMP) signaling pathway, resulting in ectopic expression of GREM1 protein in the colonic epithelium. The driver of cancer genetic testing has historically been clinical utility, based on sufficient evidence to support significant changes in patient and/or family screening and risk management recommendations (Weitzel *et al*., 2011).

Lately, multigene NGS panels have been used to analyze many high- and moderate-penetrance variants (Kurian *et al*., 2014; LaDuca *et al*., 2014; Easton *et al*., 2015). These gene panels are slightly more expensive than single-gene tests. They employ the same technology as whole-exome and whole-genome sequencing methods, although they generate less information on predefined genes. Compared to single-gene tests, cancer panels are time- and cost-efficient in cases with: 1) substantial genetic or locus heterogeneity, 2) a high prevalence of actionable mutations in one of several genes, 3) difficulty in predicting the mutated gene on the basis of phenotype or family history alone, or 4) non-informative or unavailable family history (*e.g*., adoption) (Domchek *et al*., 2013).

Slavin *et al*. (2015) showed interesting results from 348 commercial multigene panels that had been ordered during nine months in a reference cancer genetics clinics in the USA. They found that if only high risk genes had been included in the panels (*e.g. BRCA1, BRCA2, MSH6, PMS2, TP53, APC, CDH1*), the results would have been positive only 6.2% of the time, instead of 17%. Moreover, variants with unknown significance (VUS) would have been identified in 42% of the time, probably because of the inclusion of more genes in the testing, as well as the lack of knowledge on genetic variation.

In March 2013, the American College of Medical Genetics and Genomics (ACMG) issued a set of recommendations regarding the reporting of incidental or secondary findings from NGS (*i.e*., findings unrelated to the initial indication for NGS testing). When performing whole-exome or whole-genome sequencing for any clinical indication, the ACMG recommends the analysis of 56 specific genes, selected based on clinical evidence. These genetic mutations are pathogenic variants resulting in a high risk of developing a severe disease that would be preventable if identified early. Germline mutations in 16 of these genes cause hereditary cancer syndromes (Table 2).

**Table 2:** ACMG list of hereditary cancer syndromes, most with childhood onset, for reporting incidental findings.

Syndrome	Gene	Inheritance
Li-Fraumeni	*TP53*	AD
Peutz-Jeghers	*STK11*	AD
Familial adenomatous polyposis	*APC*	AD
Von-Hippel Lindau	*VHL*	AD
Multiple endocrine neoplasia	*MEN1* (type 1); *RET* (type 2)	AD
Hamartomatosis	*PTEN*	AD
Retinoblastoma	*RB*	AD
Paraganglioma-pheochromocytoma	*SDHAF2, SDHB, SDHC, SDHD*	AD
Tuberous sclerosis complex	*TSC1, TSC2*	AD
Neurofibromatosis type 2	*NF2*	AD
WT1-related Wilms tumor	*WT1*	AD

AD, autosomal dominant.

The release of these recommendations resulted in a debate regarding whether or not the analysis of these genes should be "mandatory," or whether patients should be able to opt out of such analysis and reporting (Greem *et al*., 2013). In 2015, the ACMG updated its recommendations based on the general consensus that patients should be able to opt out of the analysis of secondary findings. This decision should be made available to patients before testing, during the process of informed consent. Considering that some hereditary cancer syndromes may manifest in early childhood, these guidelines are also applied to children, whose parents will have to make the decision of opting out or not (ACMG Board of Directors, 2015).

Some challenges remain to be addressed before NGS can be used in clinical care. First, existing pretest counseling and informed consent models were not designed to address the challenges posed by multiplex testing. New educational and operational approaches must be developed to ensure that patients and families fully understand the risks and benefits of choices regarding these new tests. Second, the optimal management of carriers of moderate-penetrance mutations remains incompletely defined. Testing of presymptomatic individuals for moderate-penetrance genes does not provide the same clarity of guidance as testing for high-penetrance genes, even when a mutation is known to be present in a family. Third, there is always the potential risk of identifying VUS. Such identification complicates data interpretation and commonly requires further investigation. The frequency of this occurrence is higher with NGS compared to single-gene testing, and the management of patients with mutations of unknown significance is unclear. Finally, many syndromes have locus heterogeneity, incomplete penetrance and a high phenocopy rate, which can add to the difficulty in the interpretation of sequence data.

Furthermore, multigene hereditary cancer panel testing can lead to unexpected and complex findings. This stresses the importance of appropriate pre-test counseling and informed consent by a knowledgeable genetics professional (Fecteau *et al*., 2014). Choosing a phenotype-specific panel with high clinical utility/risk genes instead of pan-cancer panels inclusive of many "off-phenotype" and low risk genes can decrease the amount of incidental and uncertain results. If ambiguity is found on genetic testing, many additional resources are available to better clarify counseling and management for the patient and family. If an uninformative result has been disclosed, or if mutations in low and/or moderate risk genes have been found, empiric risk models may aid clinicians to offer a proper clinical management. Nevertheless, the addition of many moderate to low risk genes on panels can make it challenging to develop individualized management when a pathogenic mutation is revealed, since the phenotypic spectrum and penetrance are poorly defined so far (Slavin *et al*., 2015).

In summary, the traditional HCRA process is based on patient evaluation, considering information such as the age at diagnosis, disease histology and a detailed family history. After appropriate evaluation and consent, single-gene testing is performed, usually serially, for the most likely cancer susceptibility genes. Single-gene testing is more directed than panel testing, and genes that are unlikely to be mutated are not analyzed. The pretest counseling process for each gene allows the patient to decide whether or not to pursue a particular test after considering the clinical and personal utility of the result. Nevertheless, serial testing is a time-consuming and expensive approach for reimbursement, in addition to being cumbersome and stressful for the patient. Hence, patients often prefer panel testing due to the shorter turnaround times. Consenting procedures and counseling must, however, be adjusted to this reality.

NGS technologies now allow the simultaneous analysis of multiple genes in a cost- and time-effective manner. However, databases of genes with high- and moderate-risk are often not population-specific, with non-Caucasian populations often being under-represented. This population effect may lead to difficulties, or even misinterpretations of the results. Thus, collaborative epidemiology projects are critical to accumulate the large numbers of patients necessary to assess risk across multiple populations and inform clinical management properly. Undoubtedly, challenges will be even greater with the application of both whole-exome and whole-genome sequencing in HCRA in developing countries such as Brazil. Thus, it is crucial to proceed cautiously, in order to provide patients and their families with the best decision-making process and the access to the breakthroughs of the NGS era.
